# Histiocytic necrotizing lymphadenitis (Kikuchi-Fujimoto disease) after laparoscopic Roux-en-Y gastric bypass for morbid obesity: a case report

**DOI:** 10.1186/1752-1947-6-340

**Published:** 2012-10-08

**Authors:** Juan Garcia-Arnes, M Rosa Bernal-Lopez, Jose Luis Gallego-Perales, M Luz Vazquez-Camuñas, Ricardo Gomez-Huelgas

**Affiliations:** 1 Endocrinology and Nutrition Department, Hospital Regional Universitario Carlos Haya, Malaga, Spain; 2 Endocrinology Department, Biomedical Research Laboratory, Hospital Virgen de la Victoria, Malaga, Spain; 3 Ciber Fisiopatología de la Obesidad y Nutrición (CB06/003) Instituto de Salud Carlos III, Madrid, Spain; 4 General Surgery Department, Hospital Regional Universitario Carlos Haya, Malaga, Spain; 5 Pathology Department, Hospital Regional Universitario Carlos Haya, Malaga, Spain; 6 Internal Medicine Department, Hospital Regional Universitario Carlos Haya, Malaga, Spain

## Abstract

**Introduction:**

Kikuchi-Fujimoto disease, or histiocytic necrotizing lymphadenitis, is a rare, benign, autoimmune condition characterized by lymphadenopathy, fever and neutropenia. It is a self-limited condition of unknown etiology.

**Case presentation:**

We report the case of a 45-year-old Caucasian man with the first known case of Kikuchi disease associated with dramatic weight loss after bariatric surgery.

**Conclusion:**

Although the association between Kikuchi disease and bariatric surgery may be entirely coincidental, we speculate whether the immune dysfunction associated with weight loss may have played an etiologic role in this process.

## Introduction

Histiocytic necrotizing lymphadenitis (HNL), also known as Kikuchi or Kikuchi-Fujimoto disease, is a benign self-limiting disease of unknown cause, characterized by benign lymphadenopathy with associated fevers and systemic symptoms [1]. The differential diagnosis with other types of necrotizing lymphadenitis, such as systemic lupus erythematosus (SLE), malignant lymphoma or pyogenic infections, is established by identifying characteristic pathologic features from a lymph node biopsy specimen: paracortical necrosis with karyorrhectic foci of histiocytes, plasmacytoid monocytes, immunoblasts, and small and large lymphocytes with a predominance of T cells cluster of differentiation (CD) 8+. The pathogenesis of HNL remains controversial; it may be the result of a local T-cell-mediated hyper-response to a variety of nonspecific stimuli, mostly viral infections [2]. Alternatively, macrophages may infiltrate to repair the lymphoid tissue injured by cytotoxic T cells [3]. We describe the case of a patient who developed HNL after a laparoscopic Roux-en-Y gastric bypass (RYGB) for morbid obesity. To the best of our knowledge, this is the first such case reported in the literature.

## Case presentation

A 45-year-old Caucasian man presented with a three-week history of a tender, slow-growing, left axillary lump, associated with low-grade fever (37.5°C to 38°C) for the last week. He had hypertension and was being treated with lacidipine 4mg/day, lisinopril 10mg/day and hydrochlorothiazide 12.5mg/day. He smoked 30 cigarettes daily. Two years earlier he had undergone a laparoscopic RYGB for morbid obesity. Since that time he had lost 112kg and his body mass index had fallen from 64.6kg/m^2^ to 28kg/m^2^. He was receiving vitamins A, D and E, vitamin B complex, calcium gluconate, cholecalciferol and iron supplements. Our patient reported no family history of lymphoproliferative or autoimmune disease.

On examination, he had a tender, non-mobile, 3×4cm lymphadenopathy in his left axillary region. His physical examination was otherwise unremarkable. Laboratory studies (complete blood count, erythrocyte sedimentation rate, coagulation tests, blood glucose, creatinine, uric acid, sodium, potassium, lipids, liver parameters, cytokeratin, lactate dehydrogenase, ferritin, serum C3 and C4, antinuclear antibody, rheumatoid factor, C-reactive protein, thyroid-stimulating hormone, urine tests and serology for herpes simplex virus 1 and 2, cytomegalovirus, Epstein-Barr virus, hepatitis B and C virus, toxoplasma, human herpes virus 8, parvovirus B19, human immunodeficiency virus 1 and human T-lymphotropic virus type 1) were normal and/or negative. A thoracoabdominal computed tomography scan was normal except for the axillary lymphadenopathy.

**Figure 1:**
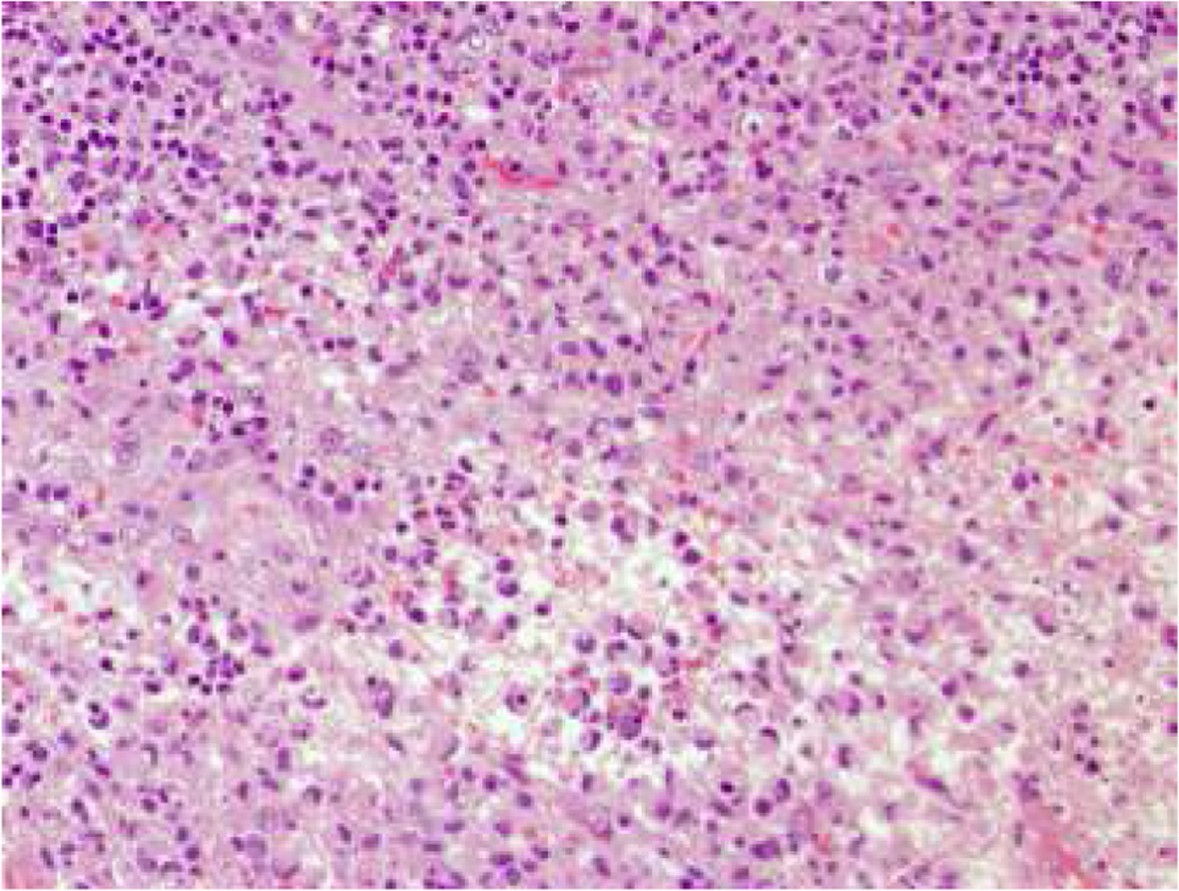
**Node biopsy (Giemsa stain).** Fibrinoid necrosis - non-neutrophilic with apoptotic bodies, cellular debris and a palisade of large lymphocytes and histiocytes with crescent nuclei resembling signet-ring cells.

A lymph node biopsy showed coagulative necrosis and frequent karyorrhexis with a lymphoplasmacytoid infiltrate (Figure 1). An investigation for mycobacteria was negative. This histological pattern is characteristic of HNL. No therapy was given and one year later our patient was asymptomatic and had experienced spontaneous complete resolution of his axillary lymphadenopathy. After four years of follow-up, our patient remains asymptomatic.

HNL is a disease with a world-wide distribution that commonly affects young adults (in their 30s), and more often affects women. The most frequent symptoms are painful lymphadenopathy, primarily involving the cervical lymph nodes, low-grade fever and malaise; arthromyalgias, cutaneous rash, sweating and splenomegaly are infrequent. Laboratory findings are nonspecific. Leukopenia is present in 50% of cases, sometimes with atypical lymphocytes.

This entity should be differentiated from other lymphoproliferative, autoimmune and postinfectious causes of lymphadenopathy. The diagnosis is established by identifying characteristic pathologic features from a lymph node biopsy: paracortical necrosis with karyorrhectic foci of histiocytes, plasmacytoid monocytes, immunoblasts, and small and large lymphocytes with a predominance of CD8+ T cells. Pathological examination enables HNL to be differentiated from other types of necrotizing lymphadenitis, such as SLE, malignant lymphoma or pyogenic infections [1,4].

The pathogenesis of HNL is unknown but immune factors seem to be important. Some cases of HNL are associated with immune diseases such as SLE, adult Still's disease, Hashimoto thyroiditis or subacute lymphocytic thyroiditis. Histological, ultrastructural and immunohistochemical findings support a hyperimmune reaction as the pathogenic mechanism of HNL.

HNL might represent a T-cell-mediated hyperimmune response to several nonspecific stimuli in genetically susceptible people. The incidence of certain human leukocyte antigen class II genes (DPA1*01 and DPB1*0202 alleles) is significantly more frequent in Japanese patients with HNL [5]. Viral agents (human herpes virus 6 and 8, Epstein-Barr virus, parvovirus B19, human T-lymphotropic virus type-1), bacteria and protozoa have all been implicated in the etiology but the supportive data are controversial [2]. Physicochemical factors (pacemaker implantation, silicone breast implant) have been anecdotally related with HNL [6].

The prognosis is excellent. A spontaneous favorable outcome within a few months is the rule. A short course of steroid therapy may be indicated if HNL is very symptomatic. However, patients must be followed-up over the long term because SLE can develop several years after the diagnosis of HNL [4].

Bariatric surgery is currently the only curative option for morbid obesity and the number of people living with bariatric surgery has increased exponentially since the mid-1990s. However, we are still not aware of all the long-term side effects associated with this procedure.

In this case, we report what we believe to be the first case of HNL after bariatric surgery. Nevertheless, we cannot discount the existence of other cases of HNL that did not have a pathologic diagnosis and may not have been reported because their courses were only mildly symptomatic and resolved spontaneously.

The association between HNL and bariatric surgery may be casual, but we cannot discard an immunological reaction secondary to RYGB. The effect of bariatric surgery on the immune system is unclear. It is well known that starvation can suppress the immune function, and partial lipectomy in rodents impairs humoral immunity [7]. Some data suggest that weight loss induced by bariatric surgery can modulate the immune system. The majority of studies addressing the effect of bariatric surgery on immune function have focused on inflammation markers. After RYGB, there is a decline in the levels of inflammatory cytokines such as interleukin (IL)-6 [8], IL-18 and tumor necrosis factor alpha [9]. Additionally, an increase in blood monocytes and a reduction in circulating dendritic cells after RYGB have also been reported [10]. Other authors have found a reduction in CD95 expression, a molecule involved in the induction of apoptosis, and an increase in CD62L (L-selectin) expression markers one year after RYGB [11]. In another study, levels of monocyte chemoattractant protein-1 and interferon gamma, which are important components of the immune response to infectious pathogens, were restored after weight loss induced by RYGB [12]. Furthermore, natural killer cell activity, innate immune cells involved in the control of cancer and infections, was enhanced after RYGB [13]. Recently, an inverse relationship between the change in certain T cells (CD4+ and CD3+) and the amount of weight lost after gastric bypass surgery has been noted [14]. Finally, bariatric surgery leads to strongly reduced leptin levels and this hypoleptinemic state may impair the immune system and increase the postoperative complications of bariatric surgery [15].

## Conclusion

Although the association of HNL and RYGB may be entirely coincidental, we cannot rule out that a hyperimmune response to extreme weight loss after bariatric surgery may lead to the development of HNL in predisposed patients.

## Consent

Written informed consent was obtained from the patient for publication of this case report and accompanying images. A copy of the written consent is available for review by the Editor-in-Chief of this journal.

## Competing interests

The authors declare that they have no competing interests.

## Authors’ contributions

JGA researched the data, wrote the manuscript and contributed to the discussion. MRBL wrote the manuscript, contributed to the discussion and reviewed the manuscript. JLGP performed the bariatric surgery. MLVC performed the histological examination of the node biopsy. RGH wrote the manuscript, contributed to the discussion, and reviewed and edited the manuscript. All authors read and approved the final manuscript.
